# Contraception and fertility transition in AMHARA National Regional State of ETHIOPIA: an application of BONGAARTS’ model

**DOI:** 10.1186/s40738-017-0039-8

**Published:** 2017-09-05

**Authors:** Nega Mihret Alazbih, Getachew Nibret Tewabe, Tariku Dejene Demissie

**Affiliations:** 1 Department of Population Studies, University of Gondar, Gondar, Ethiopia; 2 Department of Population Studies, University of Gondar, Gondar, Ethiopia; 3 Center for Population Studies, Addis Ababa University, Addis Ababa, Ethiopia

**Keywords:** Proximate determinants, Fertility, Trends, Decomposition, Bongaarts’ model, Amhara, Ethiopia

## Abstract

**Background:**

The overall decline of fertility in Amhara National Regional State between 2000 and 2011 was the highest in Ethiopia. The aim of the present study was to determine the most significant proximate determinant of fertility change during the last decade in the region using Bongaarts’ model.

**Methods:**

The sources of data were the 2000, 2005, and 2011 Ethiopia Demographic and Health Surveys. The model indices were calculated for each survey. Decomposition of fertility change into components of proximal determinants was also carried out. An index value close to 1 is a negligible inhibiting effect while a large inhibiting effect when the value very closes to 0.

**Results:**

The fertility-constraining effect of contraception increased from 0.93 in 2000 to 0.65 in 2011; however, it was lower than the effect of postpartum insusceptibility at all given times. The index of marriage remained unchanged in constraining fertility over the period (0.71 in 2000 and 0.70 in 2011) while the influence of postpartum insusceptibility slightly declined from 0.49 in 2000 to 0.54 in 2011 but was stronger than contraception and marriage. The contribution of contraception was most important in urban areas (0.46 in 2011 from 0.52 in 2005 and 0.64 in 2000); however, in rural areas, it became an important determinant over the period (0.95 in 2000 and 0.69 in 2011). The effect of postpartum insusceptibility in rural areas showed a decreasing trend (0.48 in 2000 and 0.53 in 2011). The index of marriage in rural areas was stable overtime (0.75 in 2000 and 0.73 in 2011) while in urban areas the effect declined from 0.42 in 2000 to 0.65 in 2011. Marriage was the most important proximate determinant of fertility among women with secondary and above education but the impact declined during the period (0.41 in 2000 and 0.61 in 2011). The importance of postpartum insusceptibility in limiting fertility among women with secondary and above education declined overtime (0.77 in 2000 and 0.87 in 2011) whereas the contribution of contraception became more important (0.44 in 2000 and 0.35 in 2011).

**Conclusions:**

An increase in the level of contraceptive use and effectiveness overtime was the single most important contributing factor for the recent fertility decline in the region.

## Background

Fertility levels and trends in the world generally and in a country particularly vary across time. The casual factors are diverse, but they may be broadly grouped into direct or proximate determinants and indirect or background factors [1]. The proximate determinants are bio-behavioural mechanisms that act to reduce fertility and are popularly labelled as intermediate variables. Bongaarts defined eight proximate variables impacting the fertility of a population: the proportion of women of reproductive age married in the exposure group; contraception and induced abortion fall under the second group; the third group included lactational infecundity, frequency of intercourse, sterility, spontaneous intrauterine mortality, and duration of the fertile period [2]. And, from these eight, Bongaarts identified four variables (viz., the proportion married women, prevalence of contraceptive use and effectiveness, duration of postpartum amenorrhea, and prevalence of induced abortion) as the most important proximate variables because of their higher and more direct inhibitory effect on fertility, as together they account for nearly 96% of the variation of fertility in a population [3].

Further, Bongaarts’ model has quantified the contribution of these four factors to the observed fertility level. This resulted in a simple but powerful model for analyzing how fertility changes overtime or differs from one group to another; therefore, it is the most extensively used models in the world to measure the extent to factors reduce fertility. The proximate determinants model is mostly used to identify the main factors behind fertility changes, the changes associated with fertility through time, and to compare changes in fertility among countries or regions of a country [4].

The National Population Policy of Ethiopia was adopted in 1993 with an overall goal of harmonizing the rate of population growth and economic development primarily addressing the high fertility. The policy in particular, aims to achieve a total fertility rate of 4.0 children per woman in 2015. The policy envisages meeting this target by employing different strategies such as expanding clinical and community-based contraceptive distribution services; promoting breastfeeding as a means of increasing the time span between pregnancies; and raising the minimum age at marriage for girls from the current lower age limit of 15 to, at least, 18 years [5]. In addition, the Health Policy emphasizes the need to improve the coverage and quality of family planning services in the country [6] and the Women Policy also acknowledges the need to ensure women’s access to family planning and other reproductive health services as one of the strategies to empower women [7]. The National Reproductive Health Strategy stipulates as one of its goals to reduce unwanted pregnancies and to enable individuals to achieve their desired family size [8]. The Health Extension Program involves the delivery of family planning information and services to the community through home visits [9]. The Family Law, revised in 2000, put 18 years of age as the legal marriage age for both men and women [10].

With a population of nearly 98.1 million in 2015, Ethiopia is the second most populous country in Africa next to Nigeria and is projected to be 165.1 million in 2050 [11]. The population grows at a rate of 2.6% per annum [12]. The vast majority (84%) of the population in the country lives in rural areas and is heavily dependent on rain-fed agriculture. Women of reproductive age (15–49) constitute one-quarter of the total population of the country and about 48% of the female population [12]. The main feature of fertility in Ethiopia is that it was at its highest levels at the end of the twentieth century, and that the rural-urban differentials are the highest in Africa. The total fertility rate of the country was declined from 6.6 children per woman in 1990 to 5.5 children per woman in 2000 and 5.4 children per woman in 2005 [13–15]. This shows that in the fifteen-year period, since 1990, the total fertility rate declined by only one child per woman, and stagnated between 2000 and 2005. In particular, there was a slight difference between the 2005 and 2011 total fertility rate (4.8 children per woman) [15, 16].

Fertility declined in all regions of the country except Afar, Somali, and Benishangul-Gumuz National Regional States during the last decade. It remained constant in Afar National Regional State (4.9 vs. 5.0 children per woman) and Benishangul-Gumuz National Regional State (5.4 vs. 5.2 children per woman) between 2000 and 2011. However, in Somali National Regional State the total fertility rate increased from 5.7 children per woman in 2000 to 7.1 children per woman in 2011 [14–16]. In Amhara National Regional State, however, had the greatest decline in the total fertility rate (5.9 vs. 4.2 children per woman) during the last decade as compared to Oromiya National Regional State (6.4 vs. 5.6 children per woman), Southern Nation Nationalities and Peoples National Regional State (5.9 vs. 4.9 children per woman), and Tigray National Regional State (5.8 vs. 4.6 children per woman) [14–16]. The onset of fertility decline in Amhara National Regional State has engendered much interest since it has been suggested that the fertility transition in the region is different in certain important aspects from that experienced in the past elsewhere in the country.

The available studies on proximate determinants of fertility in Ethiopia were carried out at a national level of aggregation [17, 18]; the results might have been affected by problems of aggregation bias. Since the contribution of components of proximate determinants of fertility vary from area to area, macro-level analysis rarely take into consideration of region-specific factors. In addition, these studies provide only estimation of predicted fertility levels and do not attempt to investigate socioeconomic differentials and trends in the fertility change overtime. Moreover, they do not explain the decomposition of fertility change into components of proximal determinants. As these researche gaps, there are two dimensions in applying the proximate determinants model in this study. First, keeping the regional difference and heterogeneous society in mind, the region-specific proximate determinants are the focused of this study because of the lack of a comprehensive analysis of trends in proximate determinants of fertility at the regional level in the country. Secondly, previous research lacks decomposition analysis; the present study emphasizes analysis of the trends in the fertility change between the two time points to determine contributions of the four proximal determinants. Therefore, the aim of this study was to determine the relative contribution of each of the proximal determinants in inhibiting fertility during the last decade in Amhara National Regional State, Ethiopia using the Bongaarts’ Proximate Determinants of Fertility Model.

Detailed study on the trends in proximate determinants of fertility change is important for assessing the implementation of population/reproductive health policies and family planning programs and the efforts achieving the overall goal of changing fertility behaviour, that is, increasing contraception, reducing unmet need for family planning, and rising age at first marriage. In addition, the decomposition of proximate determinants is also a basis for future projections of changes in the overall level of fertility which may be expected as a result of changing socioeconomic, demographic, cultural, institutional, psychological, health, and environmental conditions.

## Methods

### Data sources

The 2000, 2005, and 2011 Ethiopia Demographic and Health Surveys were the data sources for this analysis. They were designed to provide population and health indicators at the national and sub-national levels. The sample was selected using a stratified two-stage cluster design. Enumeration areas were the sampling units for the first stage and households comprised the second stage of sampling. A complete listing of households was carried out in each of the selected enumeration areas and a representative sample of households was selected proportionally. All women of reproductive age found in the selected households were interviewed. A representative sample of 2059, 2158, and 2163 households were selected for the 2000, 2005, and 2011 survey, respectively, in Amhara National Regional State. The 2000, 2005, and 2011 Ethiopia Demographic and Health Surveys successfully interviewed 3820, 3482, and 4433 women of reproductive age, respectively, in the region.

### Analysis

Bongaarts proposed the proximate determinants of fertility model at a time when it was assumed there was very little intercourse outside of marriage. Hence, all of the calculations he proposed were based only on currently married women of reproductive age. Bongaarts initially proposed the proximate determinants framework; social and cultural mores have been shifting to greater or lesser degrees across the world and girls and women are now having sexual intercourse before and outside marriage. Stover proposed updating the framework to account for the fact women are having sex before and outside marriage and these same women may use contraceptives and seek induced abortions [4]. This study was based on Bongaarts’ model because in most societies of the region births out of wedlock were not accepted and virginity was a prerequisite for marriage. In addition, the median age at first marriage of women of reproductive age in 2000, 2005, and 2011 was about 14.5, 14.4, and 15.1 years, respectively, while the median age at first sexual intercourse of the 2000, 2005, and 2011 was about 14.5, 14.7, and 15.3 years, respectively. That is, age at first marriage is synonymous with age of entry into sexual relations in the region [14–16]. For these reasons, we opted to conduct the proximate determinants analysis using currently married women of reproductive age.

In addition, when contraceptive use is concentrated at later ages, the Bongaarts’ model is a poor estimator of the fertility-inhibiting effect of contraception because women use contraception to stop rather than to space births [19]. These problems, however, seem to be minimal in Amhara National State where women use contraception at all ages and mainly for spacing purposes. In the region, the age pattern of modern contraceptive use was similar across the three surveys. It increased largely up to the age group of 30–34 and then declined rapidly towards the ends of the age cohort [14–16].

A) Role of proximate determinants of fertility

The model indices were calculated for each survey separately. The model expresses the total fertility rate as a product of four indices (marriage, contraception, postpartum insusceptibility, and induced abortion) and total fecundity rate [2, 3, 20]. Mathematically, it can be expressed as:

1 $$ \mathrm{TFR}=\mathrm{TF}\ast {\mathrm{C}}_{\mathrm{m}}\ast {\mathrm{C}}_{\mathrm{c}}\ast {\mathrm{C}}_{\mathrm{a}}\ast {\mathrm{C}}_{\mathrm{i}} $$

Where, *TFR* is the total fertility rate, *TF* is the total fecundity, *C*_*m*_ is the index of marriage, *C*_*c*_ is the index of contraception, *C*_*a*_ is the index of induced abortion, and *C*_*i*_ is the index of postpartum insusceptibility.

Each index has a value between 0 and 1. When the index is close to 1, the proximate determinant has a negligible inhibiting effect on fertility, whereas, when it takes a value very close to 0, it has a large inhibiting effect [2, 3]. That is, the complement value of index represents the proportionate reduction in fertility attributable to the fertility-inhibiting determinant (i.e., the lower the index value, the greater the fertility reducing impact).

The effect of abortion on fertility could not be assessed in this analysis because of a non-availability of data from the Ethiopia Demographic and Health surveys. Data on induced abortion are very rare in Africa due to illegality of induced abortion unless performed to save the mother’s life [21]. Before 2005, abortion in Ethiopia was permitted only in the case where the life of the woman was physically at risk. With the 2005 revised abortion law, abortion is allowable due to fetal impairment or deformity, rape or incest (based on the woman’s complaint only), minors (stated maternal age < 18 years), and physically and mentally unable to care for the would-be born child [22].

i) Total fecundity (*TF*)

The total fecundity is a hypothetical or potential value (the theoretical maximum level of fertility) that the total fertility rate would take if all the indices of the proximate determinants were 1. That means, a fertility level that would prevail if all women age 15–49 were married, there was no contraception use in the population concerned, no postpartum insusceptibility (beyond a maximum of 1.5 months), and no induced abortion [2, 3]. According to Bongaarts and Potter, the total fecundity of most populations falls within the range of 13 to 17 births per woman with an average of approximately 15.3 [2]. Therefore, multiplying all of the indices with 15.3 as the maximum number of births produces a predicated total fertility rate of the population.

ii) Index of marriage (*C*_*m*_)

The index of marriage represents the proportion by which total fertility rate is smaller than the total marital fertility rate as a result of marital pattern (the effect of non-marriage in terms of a reduction in fertility per women). The higher the level of marriage in the population, the less the inhibiting effect on fertility and vice versa. The index of marriage is calculated as the proportion of total fertility rate over the sum of age-specific marital fertility for a given population at a given time, that is,

2 $$ {\mathrm{C}}_{\mathrm{m}}=\Sigma \mathrm{m}\left(\mathrm{a}\right)\mathrm{g}\left(\mathrm{a}\right)/\Sigma \mathrm{g}\left(\mathrm{a}\right) $$

Where, *C*_*m*_ is the index of marriage, *m(a)* is the age-specific proportions of women currently married and is produced by dividing the number of married women of a particular age group by the number of women in the same age group and *g(a)* is the age-specific marital fertility rate and computed by dividing the age-specific fertility rate by the proportion of women currently married in each age group.

iii) Index of contraception (*C*_*c*_)

The index of contraception describes the effect of contraception on reducing the risk of conception (effect of contraception on marital fertility assuming induced abortion is absent). In terms of the marital fertility rate, the index of contraception gives the proportion by which the total marital fertility rate is smaller than total natural fertility (fertility in the absence of any deliberate birth control practice). However, the contraception index value depends on the current contraceptive prevalence rate and the average use effectiveness of the contraception. The index of contraception varies inversely with the contraception prevalence and use effectiveness of modern contraception practiced by couples. The index of contraception is calculated as the proportion of currently married women of reproductive age currently using specific methods of modern contraception, that is,

3 $$ {\mathrm{C}}_{\mathrm{c}}=1.00-\left({1.08}^{\ast }\ {\mathrm{u}}^{\ast }\ \mathrm{e}\right) $$

Where, *C*_*c*_ is the index of contraception, *u* is the contraceptive prevalence among married women (including those living together as married) age 15–49, and *e* is the average effectiveness of contraception. The constant 1.08 given in Eq. (3) is an adjustment factor designed to remove infecund women from the equation so the contraception index would become zero if effective prevalence reached 92.5%, and the remaining women are assumed to be infecund [2].

The value of *e* is estimated as a weighted average of the method specific use effectiveness (*e(i)*) level, the weights being the proportion of women employing a specific method (*u(i)*), that is,

4 $$ \mathrm{e}=\Sigma \mathrm{e}{\left(\mathrm{i}\right)}^{\ast }\ \mathrm{u}\left(\mathrm{i}\right)/\mathrm{u} $$

The indices of use effectiveness proposed for particular contraceptives are pill = 0.90, Intrauterine Contraceptive Device = 0.95, sterilization = 1.00, condom = 0.62, and others = 0.70 [2].

iv) Index of postpartum insusceptibility (*C*_*i*_)

The index of postpartum insusceptibility is intended to describe the effects of breastfeeding or abstinence on fertility in the population. According to Bongaarts and Potter, in the presence of breastfeeding and postpartum abstinence, the average birth interval equals, approximately 18.5 months (7.5 + 2 + 9) plus the duration of postpartum insusceptibility. The constant 20 in Eq. (5) represents the average birth interval length (in months) if neither breastfeeding nor postpartum abstinence is practised [2]. Therefore, the index of postpartum insusceptibility is estimated as the ratio of the mean birth interval without postpartum insusceptibility to that with postpartum insusceptibility, that is,

5 $$ {\mathrm{C}}_{\mathrm{i}}=20/\left(18.5+\mathrm{i}\right) $$

Where, *C*_*i*_ is the index of postpartum insusceptibility and *i* is the mean duration of postpartum infecundability measured in months.

B) Decomposition of Change in Proximate Determinants of Fertility

The decomposition of proportional change in the total fertility rate into the components of the proximate determinants was also carried out. The purpose of decomposition analysis of proximate determinants was to identify the sources of changes in the predicted total fertility in the last decade. A decomposition method developed by Kitagawa and later elaborated by Das Gupta was used to analyze the data [23, 24]. The decomposition was computed for three different scenarios: for the proportional change between 2005 and 2000, 2011 and 2005, and 2011 and 2000. The negative signs on the percentages indicate those proximate determinants having inhibiting effects on fertility during the period. The decomposition procedure is described below.

Step 1: Decomposition of change in total fertility rate over the time frame can be calculated as:

6 $$ {\mathrm{TFR}}_{\mathrm{t}2}/{\mathrm{TFR}}_{\mathrm{t}1}={\mathrm{C}}_{\mathrm{m}}\left({\mathrm{t}}_2\right)/{\mathrm{C}}_{\mathrm{m}}\left({\mathrm{t}}_1\right)\ast {\mathrm{C}}_{\mathrm{i}}\left({\mathrm{t}}_2\right)/{\mathrm{C}}_{\mathrm{i}}\left({\mathrm{t}}_1\right)\ast {\mathrm{C}}_{\mathrm{c}}\left({\mathrm{t}}_2\right)/{\mathrm{C}}_{\mathrm{c}}{\left({\mathrm{t}}_1\right)}^{\ast }\ \mathrm{R}\left({\mathrm{t}}_2\right)/\mathrm{R}\left({\mathrm{t}}_1\right) $$

Step 2: Proportional change for each proximate determinants and the residual between two periods

- Proportional change in total fertility rate (P_f_)

7 $$ {\mathrm{TFR}}_{\mathrm{t}2}/{\mathrm{TFR}}_{\mathrm{t}1}\hbox{-} 1 $$

b) Proportional change in total fertility rate due to a change in the index of marriage (P_m_)

8 $$ {\mathrm{C}}_{\mathrm{m}}\left({\mathrm{t}}_2\right)/{\mathrm{C}}_{\mathrm{m}}\left({\mathrm{t}}_1\right)\hbox{-} 1 $$

c) Proportional change in total fertility rate due to a change in the index of postpartum insusceptibility (P_i_)

9 $$ {\mathrm{C}}_{\mathrm{i}}\left({\mathrm{t}}_2\right)/{\mathrm{C}}_{\mathrm{i}}\left({\mathrm{t}}_1\right)\hbox{-} 1 $$

d) Proportional change in total fertility rate due to a change in the index of contraception (P_c_)

10 $$ {\mathrm{C}}_{\mathrm{c}}\left({\mathrm{t}}_2\right)/{\mathrm{C}}_{\mathrm{c}}\left({\mathrm{t}}_1\right)\hbox{-} 1 $$

e) Proportional change in total fertility rate due to a change in the remaining proximate variables (residual) - fecundability, intrauterine mortality, and sterility (P_r_)

11 $$ \mathrm{R}\left({\mathrm{t}}_2\right)/\mathrm{R}\left({\mathrm{t}}_1\right)\hbox{-} 1 $$

Step 3: Total proportional change in total fertility rate overtime due to proportional change in the proximate determinants, residual, and the interaction factor (I)

12 $$ {\mathrm{P}}_{\mathrm{f}}={\mathrm{P}}_{\mathrm{m}}+{\mathrm{P}}_{\mathrm{i}}+{\mathrm{P}}_{\mathrm{c}}+{\mathrm{P}}_{\mathrm{r}}+\mathrm{I} $$

## Results

### Biological and Behavioural characteristics of respondents (2000–2011)

Table 1 shows percentage distribution of currently married women age 15–49 by biological and behavioural characteristics in the three surveys for the region. In 2011, knowledge of at least one method of modern contraception was nearly universal among currently married women age 15–49. The prevalence of modern contraceptive methods dramatically increased since 2000 in the region, from 6.6% in 2000 to 15.7% in 2005 and 33.0% in 2011 among currently married women of reproductive age.

**Table 1:** Percentage distribution of currently married women age 15–49 by biological and behavioural characteristics in Amhara National Regional State, Ethiopia: 2000, 2005, and 2011

Variables & categories	Survey year		
	2000	2005	2011
Knowledge of modern contraception			
No	12.3	10.4	0.8
Yes	87.7	89.6	99.2
Current use of modern contraceptive method			
Non-users	93.4	84.3	77.0
Users	6.6	15.7	33.0
Modern contraceptive method-mix			
Female sterilization	0.2	0.1	0.6
Pill	3.0	3.6	1.5
Intrauterine contraceptive device	0.0	0.1	0.3
Injectables	3.4	11.7	26.5
Implants	0.1	0.1	4.0
Male condom	0.0	0.1	0.1
Exact age at first marriage			
15 years	56.5	56.8	48.3
18 years	87.0	83.0	74.9
20 years	92.1	89.9	83.0
Early marriage			
< age 18 years	87.0	83.9	75.2
≥ age 18 years	23.0	16.1	14.0
Median age at first marriage (Years)			
Urban	15.6	15.5	16.8
Rural	14.3	14.3	14.9
Total	14.5	14.4	15.1
Postpartum infecundability (Months)			
Amenorrhea	22.4	20.8	18.4
Abstinence	2.2	2.4	2.7
Insusceptibility	22.6	21.4	19.0
Number of observations	2587	2330	2776

*Source*: Calculated from 2000, 2005, and 2011 Ethiopia Demographic and Health Survey data

A skewed method-mix had been apparent in the region with only three methods, namely injectables, implants, and pills accounting for about 97% of all contraceptive use in 2011. Injectables were by far the most predominant method in all surveys. The share of injectables rose over the last decade, from 52% in 2000 to 75% in 2005 and 80% in 2011. Between 2000 and 2005 the proportion of implants to the total contraceptive use remained constant at 2% but there was an increase to 12% in 2011. In 2011 only 5% of total contraceptive use was the pill and it was 45% and 23%, respectively, in 2000 and 2005. Other methods taken together contributed to less than 3% of overall contraceptive use in 2011.

In Amhara National Regional State, the median age at first marriage among currently married women increased slightly from 14.5 years in 2000 to 15.1 years in 2011. The proportion of women who married at age 15, 18 and 20 declined from 56.5%, 87.0%, and 92.1% in 2000 to 48.3%, 74.9%, and 83.0% in 2011, respectively. The early marriage was highest in the country though it showed only a 12% decline in the last decade from 87% in 2000.

In Amhara National Regional State, the mean duration of amenorrhea declined from 22.4 months in 2000 to 18.4 months in 2011 in the total sample of currently married women. The mean duration of abstinence in the total sample of currently married women increased from 2 months in 2000 to 2.7 months in 2011. The mean duration of insusceptibility declined from 22.6 months in 2000 to 19.0 months in 2011.

### Trends and levels of predicted fertility

Table 2 depicts the model total fertility rates for each survey among currently married women age 15–49 by residence and education. Accordingly, the predicted total fertility rate of the region declined from 5.5 children per woman in 2000 to 5.1 in 2005 and further to 4.1 children per woman in 2011. Although there was an increase in the predicted fertility level in urban areas from 2.6 children per woman in 2000 to 2.8 children per woman in 2011, in rural areas the predicted fertility declined from 5.9 children per woman in 2000 to 4.5 in 2011. Women with secondary and above education had the lowest predicted fertility rates (below replacement level fertility = 2.1 children per woman) in 2011 compared with women with primary education and those who had no formal education. The model total fertility rate of women with secondary and above education was about three children lower than those with no education and about two children lower than those with primary level education.

**Table 2:** The proximate determinants indices and model total fertility rate by education and residence of women, Amhara National Regional State: 2000, 2005, and 2011

Variables & categories	Survey year											
	2000				2005				2011			
	C_m_	C_i_	C_c_	MTFR	C_m_	C_i_	C_c_	MTFR	C_m_	C_i_	C_c_	MTFR
Education												
Illiterate	0.74	0.48	0.95	5.78	0.76	0.49	0.86	5.43	0.78	0.54	0.69	4.80
Primary	0.61	0.65	0.83	4.49	0.77	0.72	0.80	5.61	0.69	0.58	0.59	3.95
Secondary+	0.41	0.77	0.44	2.35	0.57	0.90	0.46	2.24	0.66	0.87	0.35	1.86
Residence												
Urban	0.42	0.91	0.64	2.60	0.52	0.81	0.55	2.72	0.65	0.89	0.46	2.84
Rural	0.75	0.48	0.95	5.87	0.76	0.49	0.85	5.39	0.73	0.53	0.69	4.51
Total	0.71	0.49	0.93	5.50	0.73	0.50	0.83	5.12	0.70	0.54	0.65	4.20

*Source*: Calculated from 2000, 2005 and 2011 Ethiopia Demographic and Health Survey data

*Note*: *MTFR* Model Total Fertility Rate, *C*_*m*_ index of marriage, *C*_*i*_ index of postpartum insusceptibility, *C*_*c*_ index of contraception

### Changes in components of proximate determinants of fertility

The fertility-constraining effect of contraception increased as evidenced by a decrease in the index value overtime. The estimated contraception index declined from 0.93 to 0.65 during the period. The fertility-constraining effect of postpartum infecundability decreased as evidenced by an increase in its index value overtime. The estimated index of postpartum infecundability increased from 0.49 in 2000 to 0.54 in 2011. The index of marriage overtime remained unchanged with an index value of 0.71 in 2000 and 0.70 in 2011.

Table 2 shows the fertility-inhibiting effect of various proximate determinants overtime by educational status and place of residence of women. The magnitude of the influence of proximate determinants varied across time and different segments of the society in regulating fertility of a population. Of the three indices considered, postpartum insusceptibility (0.48) and marriage (0.42) showed the highest influence in inhibiting fertility in rural and urban areas, respectively, in 2000. Nonetheless, contraception constrained fertility the most in urban areas (0.46) in 2011 while postpartum insusceptibility remained the leading factor in rural areas (0.53) of the region. In addition, over the period considered, the influence of contraception in controlling the fertility of the rural women increased more than the other proximate determinants. The fertility-regulating impact of marriage in rural areas was stable overtime (0.75 in 2000 and 0.73 in 2011); on the contrary, even though the effect of marriage declined from 0.42 in 2000 to 0.65 in 2011, its impact on fertility-inhibition was higher than rural areas.

In 2000, postpartum insusceptibility and marriage were seen the primary fertility-limiting factors for illiterate women (0.48) and women with secondary and above level of education (0.41), respectively. Contraception also played a role in limiting fertility among women with secondary and above level of education (0.44) while its impact was minimal among illiterate women (0.95). A similar pattern was observed in 2005 and 2011 except the fact that contraception had gained prominence in limiting fertility among women with a level of education above primary school (0.35). Moreover, contraception showed a large shift of impact in controlling fertility among illiterate women and women with primary level of education.

### Decomposition of changes in proximate determinants of fertility

Once the indices of the three major proximate determinants had been calculated for each survey, the next step is to decompose the change in the total fertility rate observed between two time points to determine contributions of the three proximate determinants (Table 3). A decline in the total fertility rate (−25.5%) observed in Amhara National Regional State during the period 2000 to 2011 was much stronger towards the end of the period. In the period 2000 to 2005, a 6.9% decline in the total fertility rate was observed as opposed to a 19.9% decline in the total fertility rate witnessed during the period 2005 to 2011. In the first-half of the decade, 2000 to 2005, it was only the use of contraception had played a role in the decline of fertility (−10.0%) while marriage and postpartum insusceptibility were seen to have increased fertility in the region. In the second-half of the decade, an 8.0% increase in the total fertility rate was due to a change in postpartum insusceptibility while marriage and contraception were responsible for a 4.0% and 21.5% decline in the total fertility rate, respectively.

**Table 3:** Percentage impact of the proximate determinants on fertility change by education and residence, Amhara National Regional State: 2000–2011

		Education			Residence		
Period	Factors	Illiterate	Primary	Secondary and above	Urban	Rural	Total
2000–2005	Marriage	2.8	25.6	41.0	23.3	0.9	2.3
	PPI	1.4	10.7	16.7	−11.3	1.2	1.0
	Contraception	−9.9	−4.1	4.3	−15.1	−9.8	−10.0
	Interaction	−0.5	−7.2	−66.8	7.7	−0.6	−0.2
	MTFR	−6.1	25.2	−4.7	4.6	−8.3	−6.9
2005–2011	Marriage	2.1	−11.1	15.7	24.4	−3.8	−4.0
	PPI	9.9	−20.2	−2.6	10.3	9.2	8.0
	Contraception	−19.5	−26.1	−24.6	−15.1	−18.7	−21.5
	Interaction	−4.0	27.7	−5.5	−15.4	−2.9	−2.3
	MTFR	−11.6	−29.7	−17.0	4.2	−16.2	−19.9
2000–2011	Marriage	5.0	11.7	63.2	53.4	−2.9	−1.9
	PPI	11.4	−11.6	13.7	−2.1	10.5	9.0
	Contraception	−27.5	−29.1	−21.4	−28.0	−26.7	−29.3
	Interaction	−5.9	17.0	−76.4	−14.3	−4.0	−3.3
	MTFR	−17.0	−12.0	−20.9	9.1	−23.2	−25.5

*Source*: Calculated from 2000, 2005 and 2011 Ethiopia Demographic and Health Survey data

*Note*: *PPI* Postpartum Insusceptibility, *MTFR* Model Total Fertility Rate

The changes in the total fertility rate by education and residence were also decomposed by the proximate determinants. There was a consistent increase in the total fertility rate in urban areas whereas fertility in rural areas of the region had declined. The largest portion of the increase in fertility of urban areas between 2000 and 2011 happened as a result of a change in the proportion of married women of reproductive age over the period (53.4%) despite the fact that postpartum insusceptibility (−2.1%) and contraception (−28.0%) were associated with a decline in fertility. The percent change in the total fertility rate of rural women during 2005–2011 (−16.2%) was twice that observed during the first-half of the decade (−8.3%). Change in the proportion of rural women using modern contraception was responsible for a significant proportion of the decline observed in fertility (−26.7%) over the period 2000 to 2011.

From 2000 to 2005, the decline in fertility observed among women with no formal education was larger than women with above primary education. The responsible factor for this decline was a change in the prevalence of contraception over the period among illiterate women, however, the decline in fertility among women with secondary and above level of education was associated with factors that were not included in the model (interaction = −66.8%). On the contrary, the increase in fertility among women with primary level of education (25.2%) was largely attributed to the changes in the proportion of married women during the period and reduction in the duration of postpartum insusceptibility. In the second-half of the decade, 2005 to 2011, a decline in fertility in all three categories of women’s education was observed. The decline in fertility observed among women with primary level of education was the largest (−29.7%) and all the proximate determinants played a role albeit, in varying degrees. The overall decline in fertility witnessed during the 2000–2011 period by level of education of women was highest for women with secondary and above level of education (−20.9%) and least for women with primary level of education (−12.0%).

## Discussion

The predicted total fertility rate decline of 25.5% between 2000 and 2011 was decomposed into a 29.3% decline due to an increase in contraceptive practice and a 1.9% decline due to a decrease in the proportion of women in union, and a 9.0% increase due to shortening of the duration of postpartum infecundibility. The change in postpartum insusceptibility contributed the increase in the total fertility rate cancelling out a substantial part of the potential impact of contraception and contributing to a stall in fertility decline. Consistent with this finding, Nasir and Sinha reported the fertility-inhibiting effect of natural fertility control being gradually replaced by contraceptive use [25–27].

The index of postpartum insusceptibility declined in the values of its indices in the survey periods signifying a decrease in traditional cultural practices of postpartum abstinence. Contrarily, Ngalinda and Teklu et al. found an increase in the effect of postpartum infecundability in controlling fertility overtime and a primary factor for reducing the prevailing levels of fertility compared to its biological maximum [18, 28].

The model also confirmed that postpartum insusceptibility due to breastfeeding had the greatest inhibiting effect on fertility among illiterate women and women residing in rural areas. In societies where breastfeeding is generally prolonged and universal and contraceptive use is rare, the primary determinant of birth interval is the duration of breastfeeding [29–31]. However, this was not true in the urban areas where contraception played the leading role in inhibiting fertility. The general observation is that the duration of breastfeeding declines with development. In particular, breastfeeding declines with urbanisation and education [32].

The fertility-constraining power of contraception showed remarkable increase overtime. Fertility decline in Africa initially began in Kenya, Botswana, and Zimbabwe due to high levels of contraceptive practice, ranging between 27 and 44% among currently married women of reproductive age [33]. Slowing of fertility decline and contraceptive prevalence in sub-Saharan Africa have impacted on the pace of fertility transition [34–37], that is, a weakened family planning service environment is contributing to the stall in fertility decline [38, 39]. An increase in contraceptive prevalence has emerged as a dominant explanation for the observed fertility declines [40–45]. However, Ngalinda and Teklu et al. found the use of contraception had only a very minor fertility-inhibiting effect compared to its biological maximum [18, 28].

Contraception led to a remarkable change in inhibiting fertility among illiterate and rural women between 2000 and 2011. This may be due to the deployment of health extension workers since 2003 in each Kebele (the lowest administrative unit of urban-rural structure of the region and sub-divided into villages). The accompanying diffusion effects brought by such new endeavours are contributing factors in shaping the contraceptive use and fertility behaviour of the society, especially in rural areas of the region [9].

The index of marriage contributed a minor proportion of the decline in the total fertility rate in the region due to the small increase in age at first marriage, as marriage is a customary and socially established practice that has been carried on for generations in most communities of the region. However, the proportion of marriage is one of the major fertility-inhibiting factors and played increasing role overtime [18, 25, 45–49].

The predicted total fertility rate of the urban areas in the region consistently increased during the period due to a reduction in age at first marriage. In Amhara National Regional State, there has been a decline of more than two years in the median age at first marriage from 22.9 to 20.6 years in urban areas especially amongst the youngest women [14–16]. Consistent with the finding reported by Westoff where a decline of one year of age at first marriage lead to an increase of 5–6% in the total fertility rate [50], the finding of the present study also suggests a change in age at marriage in urban areas to some degree brought about the slight increase in fertility during the period in the region.

This study has a number of strengths. First, the study utilized a large population-based sample, a randomly selected population, a high response rate (>98%), the use of standardized surveys, the well-tested validated questionnaires, and the high quality of data due to extensive training of data collectors and support during the fieldwork, with concurrent data entry and editing with feedback during fieldwork. Second, calculations were done after the data were weighted for the sampling probabilities and non-responses. Third, decomposition of proximate determinants was applied to understand the sources of change in fertility overtime. Finally, the study used a five-year interval surveys and can provide important data for monitoring population/reproductive health policies and family planning programs.

Our study also has some limitations. During decomposition analysis, the index of induced abortion was taken as 1 in all populations, that is, induced abortion was assumed to have no inhibitory effect on fertility of a woman because of a lack of data in each survey. The exclusion of abortion in the model could have an effect on the estimates of total fecundity especially in areas where it is high. It is documented that induced abortion rates in sub-Saharan Africa are growing very fast, particularly in urban areas and amongst the youngest [51–57]. Another limitation of Ethiopia Demographic and Health surveys was that they employed a cross-sectional retrospective design to collect information about women’s birth. The validity, reliability and completeness of cross-sectional retrospective data depend on the respondent’s recall of past events. Differences in recall periods had been shown to influence the quality of data in relation to fertility, since individuals tend to recall the most recent period. Fertility estimations in each survey were dependent on the accuracy of the respondents’ current age and the quality of birth history data. A common feature of age distributions in each survey was heaping on ages ending with 0 and 5. The birth history collected from each woman age 15–49 is essentially a listing of all the live births the woman has had. The most important attributes of each birth, apart from the fact of the birth, are the month and year of birth; whether the child has survived to the date of interview; and, if the child has died, the age at death. The reliability of the total fertility rate estimates depends on the accuracy and completeness of reporting and recording of births and deaths. Typically, the most serious source of non-sampling errors in each survey that collects retrospective information on births and deaths arises from the omissions of children and the displacements of birthdates.

## Conclusions

An increase in the level of contraceptive use and effectiveness overtime among fertile women was the single most important contributing proximate determinant to the recent fertility decline in the region. Postpartum insusceptibility contributed to an increase in fertility due to a shortening of the duration of postpartum infecundibility during the last decade. Regardless, this increase was compensated by the fertility-inhibition effect of contraceptive use in Amhara National Regional State. Marriage contributed a little in constraining fertility in the 11-year span due to the unchanging of age at first marriage at early age (before age 18) in the region.

Although fertility declined overtime in the region, a close look at fertility experience of women disaggregated by various characteristics revealed that those who live in rural areas and had primary or no education were the ones with high fertility. Fertility transition in the region could be accelerated through significantly increasing girls’ education beyond the primary level of education and strengthening education and communication about the status of women. On the other hand, strengthening of existing reproductive health and family planning programs is required in order to increase the quality and quantity of contraceptive use and achieve higher use effectiveness which will lead to a greater contribution to fertility decline. In this regard, strengthening the Health Extension Workers, Health Development Armies and other volunteers’ community health workers could have a powerful role in reaching women with family planning messages, and impacts the ability to maintain the current impetus in contraceptive use. There is the high level of unsatisfied demand for fertility regulation and the increasing contraceptive use in the future particularly in rural areas would have a major impact. In addition, the implementation of the family law which asserts the minimum age of marriage as 18 years should be ensured by creating community awareness using social mobilizations, strengthening local enforcements, and establishing legal age assessment committees in each rural Kebeles.
